# Supplementary material for: Reconciling Mining with the Conservation of Cave Biodiversity: A Quantitative Baseline to Help Establish Conservation Priorities
Source: PLoS One. 2016 Dec 20;11(12):e0168348. doi: 10.1371/journal.pone.0168348 (PMC5173368; doi:10.1371/journal.pone.0168348)
Supplement: S1 Dataset — (ZIP) [file pone.0168348.s002.zip › Taxa/Serra Norte/SN_2013/N4WS/Lista N4WS-58.pdf]

CAVIDADE N4WS-0058							
**Classe**	**Ordem**	**Fam/Outros**	**Gên/Outros**	**Espécie**	**S-U**	**U-S**	Zon.
Annelida	Oligochaeta			sp.	3		E
Arachnida	Acari	Bdellidae		sp.1	X		E
Arachnida	Acari	Bdellidae		sp.3		X	E
Arachnida	Acari	Laelapidae	*Cosmolaelaps*	sp.1		X	P
Arachnida	Acari	Laelapidae		sp.1	X		P
Arachnida	Acari	Mesostigmata		jovem		X	E
Arachnida	Acari	Podocinidae	*Podocinum*	sp.1	X		E
Arachnida	Amblypygi	Phrynidae	*Heterophrynus*	*longicornis*	1	1	E
Arachnida	Araneae	Ctenidae		jovem	1		P
Arachnida	Araneae	Drymusidae		jovem	X	X	E
Arachnida	Araneae	Mysmenidae		jovem	X		E
Arachnida	Araneae	Ochyroceratidae	*Speocera*	sp.1	X	X	E/P
Arachnida	Araneae	Pholcidae	*Mesabolivar*	*aurantiacus*		X	P
Arachnida	Araneae	Pholcidae	Ninetinae	sp.1		X	P
Arachnida	Araneae	Theridiidae	*Theridion*	sp.2		X	E
Arachnida	Araneae	Theridiosomatidae	*Plato*	sp.1	X		E
Arachnida	Pseudoscorpiones	Chernetidae	*Spelaeochernes*	sp.1	X	X	E/P
Arachnida	Pseudoscorpiones	Chthoniidae	*Pseudochthonius*	sp.1	X	X	E/P
Diplopoda	Polyxenida	Hypogexenidae		sp.1	X	X	P
Entognatha	Collembola	Paronellidae		sp.1	X		E
Entognatha	Collembola	Paronellidae		sp.4	X		P
Entognatha	Collembola	Paronellidae		sp.6	X		E
Entognatha	Diplura	Campodeidae		sp.1	X	X	E
Entognatha	Diplura	Japygidae		sp.1	X	X	E
Insecta	Coleoptera	Scydmaenidae		jovem	X		E
Insecta	Diptera	Ceratopogonidae		jovem	X		P
Insecta	Diptera	Drosophilidae	*Drosophila*	*eleonorae*		X	E
Insecta	Diptera	Keroplatidae	*Euceroplatus*	sp.		X	P
Insecta	Diptera	Mycetophilidae	*Neoempheria*	sp.	X		E
Insecta	Diptera	Psychodidae	*Telmatoscopus*	sp.		X	P
Insecta	Diptera	Tipulidae		jovem	X		E
Insecta	Heteroptera	Cydnidae	*Pangaeus*	sp.1	X		E
Insecta	Heteroptera	Lygaeidae	Rhyparochrominae	sp.1		X	E
Insecta	Heteroptera	Miridae		jovem		X	P
Insecta	Heteroptera	Schizopteridae	Schizopterinae	sp.2		X	E
Insecta	Homoptera	Cicadellidae		sp.3		X	E
Insecta	Homoptera	Cixiidae		jovem	X	X	E/P
Insecta	Hymenoptera	Apoidea		sp.1		1	P
Insecta	Hymenoptera	Formicidae	*Apterostigma*	sp.1	X		E/P
Insecta	Hymenoptera	Formicidae	*Brachymyrmex*	sp.1	X		E
Insecta	Hymenoptera	Formicidae	*Cyphomyrmex*	sp.2	X		P
Insecta	Hymenoptera	Formicidae	*Dolichoderus*	aff. *bispinosus*		X	E/P
Insecta	Hymenoptera	Formicidae	*Nylanderia*	sp.1	X		E
Insecta	Hymenoptera	Formicidae	*Nylanderia*	sp.3		X	P
Insecta	Hymenoptera	Formicidae	*Octostruma*	sp.1	X		P
Insecta	Hymenoptera	Formicidae	*Pachycondyla*	*striata*	X		P
Insecta	Hymenoptera	Formicidae	*Trachymyrmex*	sp.2		X	E/P
Insecta	Isoptera	Rhinotermitidae	*Heterotermes*	sp.		X	P
Insecta	Isoptera	Termitidae	*Nasutitermes*	sp.	X	X	P

Insecta	Lepidoptera	Noctuoidea		sp.1		1	P
Insecta	Orthoptera	Phalangopsidae	*Phalangopsis*	sp.	11	5	E/P
Insecta	Orthoptera	Phalangopsidae		sp.1		1	P
Insecta	Psocoptera	Psocomorpha		jovem		X	E
Malacostraca	Isopoda	Armadillidae		sp.1		X	P
Symphyla		Scutigerellidae	*Hanseniella*	sp.1	X		E
Amphibia	Anura	Strabomantidae	*Pristimantis*	cf. *fenestratus*	3		E
Mammalia	Chiroptera	Phyllostomidae	*Carollia*	*perspicillata*		1	P
Mammalia	Rodentia	Cricetidae	*Rhipidomys*	sp.	1		P
